# Treating Lower Phantom Limb Pain in the Postoperative Acute Care Setting Using Virtual Reality: Protocol for a 4-Phase Development and Feasibility Trial

**DOI:** 10.2196/68008

**Published:** 2025-05-23

**Authors:** Renée El-Gabalawy, Megan Crooks, Michael Sean Dyck Smith, Elizabeth Hammond, Patrick Gross, Marinya Roznik, David Perrin, Kristin Reynolds, Gabrielle Logan, Lily Pankratz, Hilary Johnson, Linda Girling, Daniel Wiebe

**Affiliations:** 1 Department of Anesthesiology, Perioperative and Pain Medicine University of Manitoba Winnipeg, MB Canada; 2 Department of Clinical Health Psychology University of Manitoba Winnipeg, MB Canada; 3 Department of Psychology University of Manitoba Winnipeg, MB Canada; 4 Department of Psychiatry University of Manitoba Winnipeg, MB Canada; 5 CancerCare Manitoba Winnipeg, MB Canada; 6 National Research Council Winnipeg, MB Canada; 7 Department of Physical Therapy University of Manitoba Winnipeg, MB Canada; 8 Shared Health Inc Winnipeg, MB Canada; 9 Department of Surgery University of Manitoba Winnipeg, MB Canada

**Keywords:** phantom limb pain, virtual reality, lower limb amputation, graded motor imagery, feasibility, postoperative, acute, patient-centered, preventive, pre-emptive

## Abstract

**Background:**

Phantom limb pain (PLP) affects most people living with lower limb amputations (LLAs). Nonpharmacological interventions, such as graded motor imagery (GMI), have demonstrated promise as PLP treatments. However, GMI access is limited by low patient buy-in and long public outpatient wait times. Considering PLP has been shown to be more prevalent and severe immediately following LLA, there is an urgent need to bypass barriers to allow for prompt access to PLP interventions. In response to this need, the multidisciplinary research team in this study developed a virtual reality (VR) program that administers GMI treatment. This novel intervention may be completed independently and promptly within the postoperative acute care setting. Before conducting a randomized controlled trial, the VR-GMI program must be developed and refined through a rigorous and multistage feasibility assessment.

**Objective:**

This protocol aims to outline the development and feasibility of the VR-GMI prototype for treating people with LLAs in the postoperative acute care setting (ie, inpatient and home settings) through an iterative, patient-centered, and descriptive approach.

**Methods:**

Four phases of prototype development and assessment were conducted. In phase 1 (completed), the VR-GMI prototype was developed in collaboration with engineers at the National Research Council and in consultation with patient partners. In phase 2 (completed), people with lived experience with amputations were recruited from local physiotherapy and prosthetic clinics to trial the VR-GMI program and provide feedback through semistructured interviews and self-report measures. Phase 3 (completed) consisted of a descriptive case series of individuals who trialed the VR-GMI prototype immediately following their LLAs in the hospital. Results from phase 3 informed the development of a primary quantitative feasibility study. Phase 4 (underway) aims to evaluate the acceptability and pilot outcomes of the VR-GMI program in hospital and home settings as well as improve study procedures for a future randomized controlled trial (phase 4A). Iterative developments were made to the VR-GMI program between each phase to improve prototype fidelity. These iterative developments will also be reviewed in a series of focus groups to finalize the VR-GMI prototype (phase 4B).

**Results:**

Recruitment for phases 1 and 2 was completed in September 2023. Phase 3 was completed in July 2024, and phase 4A is currently underway with 15 participants recruited as of March 2025.

**Conclusions:**

The intervention developed is the first VR PLP treatment implementing GMI and prioritizing an in-depth, patient-centered approach before assessing its efficacy. Doing so will improve the likelihood of successful clinical implementation. Moreover, very few PLP interventions have been assessed in the acute postoperative period when they may prevent PLP before its onset.

**Trial Registration:**

ClinicalTrials.gov NCT06638918; https://clinicaltrials.gov/study/NCT06638918

**International Registered Report Identifier (IRRID):**

DERR1-10.2196/68008

## Introduction

### Literature Review

More than 7000 lower limb amputations (LLAs) are performed in Canada each year due to (in order of decreasing prevalence) diabetic, vascular, traumatic, cancerous, and congenital indications [1]. Approximately 70% of these individuals will develop phantom limb pain (PLP) [2,3], a type of neuropathic pain where one experiences uncomfortable kinesthetic or somatosensory sensations in their missing limb following amputation [4]. PLP can be experienced for the first time within days following amputation (if not, within 1 year) and persists to variable degrees of intensity over a lifetime depending on the individual [5]. Research suggests that PLP is more likely to occur in people with more recent amputations compared to those who underwent amputations further in the past [2,3]. In addition, those who have an amputation due to chronic conditions (eg, vascular disease or diabetes mellitus) may have worse outcomes than those who undergo traumatic amputations because of preoperative factors (eg, preamputation pain) [2]. While PLP may improve over time if untreated, the lack of PLP treatment most often can significantly reduce quality of life, both psychologically and physically [6,7]. For example, research suggests PLP reduces mobility, which has adverse effects on independence (such as maintaining employment) and social role fulfillment [6,7]. PLP has also been implicated in a variety of other negative sequelae, including poorer sleep quality and increased depressive or anxious symptomatology [8-10]. The high prevalence of PLP among the growing number of LLAs, coupled with a lack of outpatient support, reflects an urgent need to develop effective treatments tailored to address the gap in care between the inpatient and outpatient settings to mitigate these negative sequelae.

In response to this need, researchers have developed many pharmacological and nonpharmacological PLP treatments. Currently, pharmacological interventions are among the most prevalent in clinical practice in treating PLP [11,12]. However, recent studies suggest that pharmacological interventions are both ineffective compared to placebo and considered unsatisfactory by patients with PLP [10-14]. Accordingly, researchers have turned their attention to nonpharmacological PLP treatments. One such treatment, graded motor imagery (GMI) [15], directly targets the central mechanisms implicated in PLP [16] and consists of 3 stages. The first stage of GMI is left and right discrimination (also known as implicit motor imagery), which entails quickly sorting images of left and right limbs. The patient then moves to the second stage, explicit motor imagery, which involves deliberate focus on phantom limb visualization. In the third stage, the patient undergoes mirror therapy, which involves visual feedback of the reflected intact limb (in a mirror box) to create the illusion of phantom limb movement. Despite the efficacy of GMI for PLP [15,17], it is often not clinically feasible due to its heavy reliance on physiotherapists with specialized training [18]. In Manitoba (where this research is being conducted), it can take up to 2 years to visit a pain specialist from a public treatment center [19], let alone a physiotherapist who specializes in GMI. To successfully implement GMI as a PLP treatment, barriers to its access need to be mitigated.

By eliminating GMI barriers, it may be possible to prevent or mitigate PLP onset through early intervention. While most treatments are administered well after PLP first occurs (ie, >3 months following amputation) [20], researchers have successfully prevented PLP by administering both pharmacological and nonpharmacological interventions during the perioperative period of amputation [20-23]. For example, both epidural and systemic analgesia (ie, morphine and ketamine) administered before, during, and after major limb amputation have been shown to reduce PLP incidence compared to a control group up to 1 year and 6 months following the procedure, respectively [21]. Similarly, surgical intervention geared toward neuroma prevention (such as targeted muscle reinnervation) used at the same time as major limb amputation has demonstrated significant PLP reduction compared to a control group at 3-month follow-up [22]. More recently, researchers have even begun to investigate the prophylactic effects of nonpharmacological PLP interventions, which have been shown to reduce PLP incidence and intensity with mirror therapy if administered <2 months following LLA [20,23]. Administering PLP treatment early may not only bypass barriers to access, but it may also be more efficacious, including preventing the onset of PLP, which could have significant implications for negative downstream health and social sequelae.

### Purpose and Rationale

To address current barriers to PLP care, our multidisciplinary research team (consisting of clinical psychologists, physiotherapists, physiatrists, anesthesiologists, surgeons, and engineers) developed a novel virtual reality (VR) program that allows people with LLAs to self-administer GMI treatment. VR is a computer-generated 3D simulated environment in which a user may be immersed with the use of a mounted headset. In recent years, it has been used to treat PLP by simulating mirror therapy or other gamified interventions [24]. The potential advantages of integration between VR and GMI are threefold. First, research suggests VR is more motivating and usable than 2D treatment administration modalities [25,26]. The novelty offered by VR will be invaluable to administer GMI psychoeducation and tasks in a gamified manner to increase patient buy-in, knowledge, and adherence [27]. Second, all GMI psychoeducation and treatment tasks are embedded on the device, removing the need to wait for and travel to a physiotherapist. In recent studies, VR PLP (without embedded GMI) treatment has already been self-administered by participants in their homes and produced promising pilot and feasibility outcomes [28,29]. Finally, virtual visual feedback may be of particular benefit to people with LLAs. Research suggests lower limb movements are more natural when performed asymmetrically (such as pedaling or walking); therefore, VR (through its capacity for asymmetrical movement) may produce more favorable outcomes for people with LLA compared to traditional mirror therapy (which can only be used symmetrically) by generating a higher sense of agency and ownership (defined as perceived control over and possession of the virtual limb, respectively) [30-32]. By bypassing barriers to care through a novel VR-GMI program, PLP can be targeted in the perioperative setting before its onset, when PLP is suggested to be the most intense and receptive to intervention [3,20].

### Study Aims

This research aims to develop and assess the feasibility of a novel VR-GMI program for the treatment and prevention of PLP in persons with LLAs through 4 developmental and evaluative phases. The aim of phase 1 (completed) was to develop the initial prototype of the VR program through a multidisciplinary and patient-oriented approach, in accordance with GMI literature and feedback from people with LLAs. In phase 2 (completed), the perceptions of the VR prototype from people with LLAs living in the community were evaluated using a multimethod design. In phase 3 (completed), the preliminary feasibility of the VR prototype was assessed in the acute postoperative setting using a case series design. In phase 4A (in progress), a larger quantitative feasibility study is being conducted to evaluate the VR program in the acute postoperative period (including the hospital and transition to home) immediately following LLA. Phase 4B will conclude with a series of focus groups in which a subset of feasibility trial participants will discuss their experiences in the VR-GMI program and how it might be improved.

**Figure 1:**
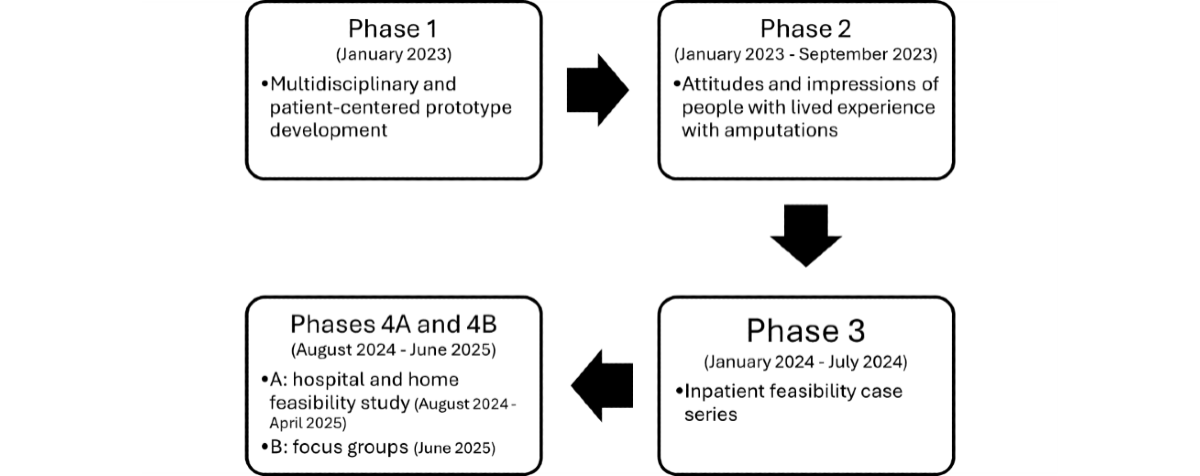
Visual overview of the 4 phases through which the feasibility of the virtual reality–graded motor imagery program will be assessed.

Each of the 4 phases was designed according to the Virtual Reality Clinical Outcomes Research Experts (VR-CORE) model [33], which outlines expert recommendations on how to validate a medical VR program. VR1 studies are patient-centered and involve collecting feedback from the population the VR program is designed for (including both clinicians and patients) to ascertain attitudes and expectations regarding treatment. To ensure the VR-GMI program reflects the needs of people with LLA, phases 1, 2, and 3 all meet the criteria for a VR1 study. Meanwhile, phase 4A meets the criteria of a VR2 study, which focuses on establishing the acceptability, feasibility, tolerability, and preliminary efficacy of the VR program. Birckhead et al [33] also describe VR3 studies, which entail using a randomized controlled trial (RCT) to assess the efficacy of a VR program. The VR3 study of the VR-GMI program will be outlined in a separate future protocol. The VR-GMI program will be finalized through a focus group (a VR1 study) to ensure the prototype is of the highest fidelity before proceeding to VR3. Figure 1 presents an overview of the 4 studies that will evaluate the feasibility of the initial VR-GMI prototype.

## Methods

### Ethical Considerations

Phase 1 did not involve participants; therefore, ethics approval was not required. Consent forms relevant for each phase were reviewed by a research assistant with participants to ensure informed consent. Any ethical amendments were immediately addressed, updated, and communicated to participants. Data are deidentified using a random computer-generated 5-item alphanumeric code. All data are stored in a locked cabinet in a locked office in a secure facility. All investigator communications were completed via password-protected files, and any paper notes (with no identifiers) were destroyed immediately after use. Any photos taken during this study received additional consent from the participants. Ethics approvals for each phase were received. Phase 2 received ethics approval from the research ethics board (Fort Garry) at the University of Manitoba (HE20220374); phase 3 received ethics approval from the research ethics board (Bannatyne) at the University of Manitoba. Phase 4 received ethics approval from the research ethics board (Bannatyne) of the University of Manitoba (B2022107). Honorariums of CAD $50 (US $38.45) were provided to study partners in phase 1, and CAD $25 (US $19.23) gift cards will be provided to participants in all other phases.

### Phase 1: Multidisciplinary and Patient-Centered Prototype Development (Completed)

#### Overview and Objective

In phase 1, the initial VR prototype was developed collaboratively within a multidisciplinary team, including engineers (MS and DS) affiliated with the National Research Council (NRC) of Canada and led by a clinical psychologist (REG) from the University of Manitoba. In addition, study partners (including people with LLAs and physiotherapists) and collaborators (anesthetists and physiatrists) were consulted to iteratively incorporate their feedback and lived experience with amputations during initial prototype development. The objective of phase 1 was to develop a virtual GMI prototype through multidisciplinary and patient-centered feedback.

#### Study Partners

The study partners consisted of 5 people with lived experience with amputations, including 2 (40%) physiotherapists (EH and PG) and 3 (60%) people with LLA. Both physiotherapists had experience working with people with LLA, using GMI (EH) or other forms of physiotherapy (PG). All 3 of the study partners (male participants: n=2, 67% and female participants: n=1, 33%) with LLAs had experienced disruptive PLP at some point following their surgeries, with the pain being reported to be the worst at its onset. Study partners who were people with LLA were given a CAD $50 (US$38.45) honorarium for their participation.

#### Procedures

**Figure 2:**
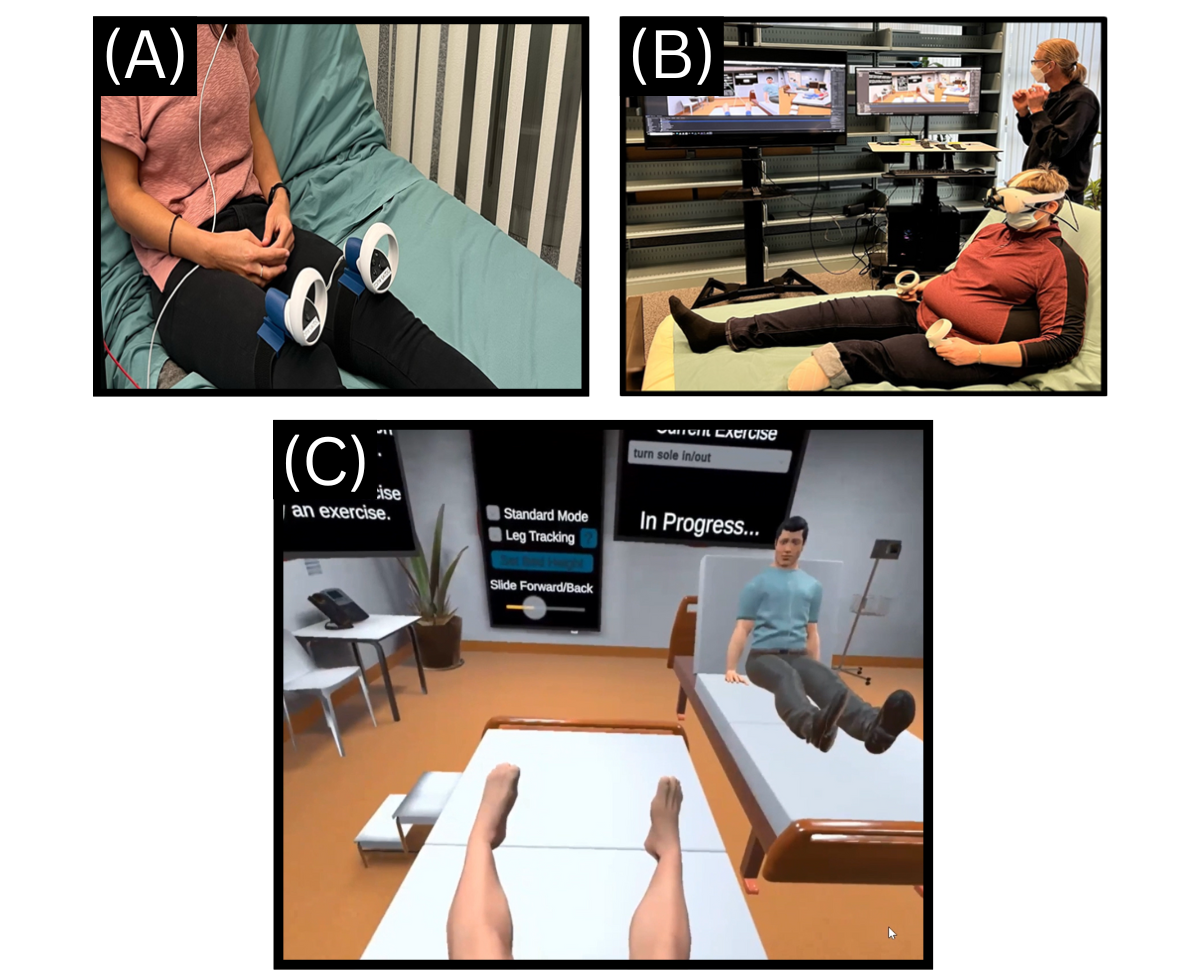
(A) Research team member trialing the hand control trackers attached to the legs via 3D-printed adapters. (B) Study partner trialing the virtual reality prototype. (C) Larger image of limb simulation stage in program.

An initial multidisciplinary team meeting was held in January 2021 to formulate a plan for prototype development. Team members then aimed to recruit 5 people with lived LLA experience. Study partners who were people with lived experience with LLAs were approached by team members in their respective clinics to ask about their interest in participation in this program of research. If they expressed interest, the potential study partners were directly contacted by study personnel to receive further information. Interested partners (n=3, 60%) participated in an initial semistructured interview in August 2021 with the principal investigator (REG), research personnel, and lead engineer via Zoom (Zoom Communications, Inc; due to the COVID-19 pandemic). Interview questions inquired about amputation history (including current and past interventions accessed for PLP), initial thoughts relating to the proposed VR prototype, and continued interest in involvement throughout development. Interviews were 1 hour in length. Partners were continually engaged in initial development via Zoom and email to provide feedback on prototype development. Once a prototype was in place, multidisciplinary team members and study partners both viewed videos of the prototype and trialed it at the NRC to provide feedback. It should be noted that, due to delays in development (due to COVID-19), the explicit motor imagery task was not evaluated by study partners over video or in person; however, the written scripts were reviewed and revised by partnered physiotherapists. In addition, one of the study partners with an LLA stopped responding to research correspondence midway through development for unknown reasons. Figure 2 shows an image of our research team and one of our partners (with respective consents provided) trialing the VR-GMI program.

#### The Initial VR-GMI Prototype

The VR-GMI program was developed using the Unity game engine (Unity Technologies) and operates on the Oculus Quest 2 (Meta Platforms, Inc), a commercially available VR hardware system with a head-mounted display and hand controllers. This stand-alone system is advantageous as it is not encumbered by wires connecting it to an external computer, allowing for ease of setup and use. It also includes functionality for the clinician and research personnel to follow along with the participant by casting over Wi-Fi through the Oculus app (or by attaching a Link cable if Wi-Fi is unavailable) a mirror image of the virtual environment as experienced by the participant to a separate display device, such as a smartphone or tablet. To increase feelings of embodiment for the participant by having their virtual leg movements follow their real-world leg movements, leg tracking was also incorporated into the system. Initial experimentation with third-party tracking devices using ultrasound and inertial measurement units was promising, but performance was inconsistent when tested in different environments. Prioritizing consistency to avoid breaking presence in VR, an alternate approach was adopted using the Oculus Quest 2 hand controllers to track the participant’s legs. Custom adapters for attaching the controllers to the user’s legs were designed and 3D printed. When leg tracking is enabled in the program, the hand controllers are strapped to the participant’s legs above the knee using custom adapters (Figure 2).

Following initial development of the base prototype, GMI elements were included with guidance from physiotherapist partners. The GMI stages, including left and right discrimination, explicit motor imagery, and mirror therapy, formed the basis of the VR program. Implementation was designed to take advantage of the unique affordances of VR. For example, the left and right discrimination task was implemented in a whimsical environment in which the user interacted with the images of feet using their controllers to increase engagement. The explicit motor imagery phase incorporated recorded audio of a body scan to enhance relaxation and focus on the phantom limb [34-36]. Meanwhile, the mirror therapy stage included asymmetrical exercises in the virtual visual feedback, a feature that is not feasible during traditional mirror therapy. Due to the capacity of VR to provide asymmetrical visual feedback, the mirror therapy stage was dubbed “limb simulation.”

#### Priorities for Future Development

Across both virtual and in-person meetings with study partners, common recommendations emerged that informed the optimization of the VR prototype. These developmental priorities included addressing technological barriers and improving leg tracking. The details of these recommendations can be reviewed in Multimedia Appendix 1.

### Phase 2: Attitudes and Impressions of People With Lived Experience With Amputations (Completed)

#### Overview

In phase 2, developmental priorities for the finalized VR-GMI prototype were assessed by collecting feedback from people with LLAs to ascertain preliminary treatment attitudes and expectations. To accomplish this, 12 people with LLAs were recruited from community settings to trial the VR-GMI program and detail their experience through a combination of observations, self-report measures, and semistructured interviews.

#### Study Design and Objectives

This study used a multimethod design to elucidate participant perspectives on the VR-GMI prototype in a single, intensive VR session. The first and primary objective was to assess anticipated barriers to and facilitators of using the VR-GMI program in the intended administration settings (including home and hospital settings immediately following LLA) through semistructured interviews. A second objective was to evaluate how engaging and immersive the VR-GMI prototype was using self-report measures. Observations and informal discussions throughout the VR sessions provided context for the triangulated data to inform program development.

#### Recruitment and Inclusion Criteria

The research team planned to recruit 10 to 15 participants via purposive sampling through community partners, including physiotherapists, surgeons, and prosthetists. Such a sample size is akin to similar user-centered studies evaluating the development of novel interventions [37,38]. Recruitment posters were disseminated in person as well as through email and institutional social media accounts. Interested individuals were contacted by the researchers directly or over the phone if the participant provided verbal consent to a community partner. Potential participants were screened over the phone for the following eligibility criteria: must have a unilateral LLA proximal to the ankle, must be aged ≥18 years, must not be experiencing any hearing or vision loss that would affect interaction with a VR headset, must have experienced PLP or sensation either in the past or present, and must agree to be audio recorded. Participants were also informed that they would be given a CAD $25 gift card (US $19.23) to Tim Hortons to thank them for participating. The research team aimed to recruit 10 to 15 participants between March 2023 and September 2023. By the end of this recruitment period, 12 participants were recruited.

#### VR Intervention Procedure

Each participant tested the VR program in an intensive session (approximately 2.5 h in duration) at the NRC building in Winnipeg, Canada. Appointments were booked via phone following eligibility screening. Gift cards were given promptly following completion of the informed consent form. Before using the VR program, each participant completed a semistructured interview to collect baseline characteristics, including amputation etiology and pain history. The research assistant (MC) then verbally administered GMI psychoeducation and technical instructions regarding the VR hardware. Afterward, the participant was instructed to vocalize all thoughts they had about each of the 3 VR GMI stages as they navigated them, including operational or instructional confusion, stylistic choices, etc. During program use, the research assistant recorded observations of the participant’s behavior on a laptop as well as any vocalizations from the participant. Approximately half of the sessions included at least 2 research personnel, with an engineer at most of the sessions.

Following each of the 3 VR stages, the participants doffed the headset to discuss their experience in that particular task. The research assistant also clarified any observations made during that stage. Once the participants completed the entire VR program, they filled out a series of self-report measures to gauge the quality of their experience in the VR program, in the following order:

- The User Engagement Scale [39] is a 31-item questionnaire that measures how rewarding a given user experience was. It was rated on a 5-point Likert scale and validated in the head-mounted VR context [40].
- The Presence Questionnaire [41] is a validated 20-item questionnaire that measures how immersive a virtual experience was. Each item was rated on a 7-point Likert scale.

Participation concluded with an exit interview discussing anticipated barriers to and facilitators of using the VR program. The final exit interviews were audio recorded via Trint for later transcription and analysis.

### Statistical Analysis

Given the small sample size and nature of this study, scores across various measures were descriptively analyzed using R software (version 4.3.1; R Foundation for Statistical Computing). Meanwhile, exit interview transcripts were coded line by line and examined using reflexive thematic analysis [42,43]. Following separate qualitative and quantitative analyses, the results were integrated, along with observations, for interpretation and extrapolation of developmental priorities for the VR-GMI program.

### Phase 3: Inpatient Feasibility Case Series (Completed)

#### Overview

In phase 3, the VR-GMI prototype was implemented in the acute postoperative hospital setting immediately following LLA. Given the novelty of this methodological approach, the research aimed to understand participant experience and feedback on the intervention as well as identify barriers encountered during recruitment and administration before the initiation of a larger feasibility trial. The information gathered directly impacted modifications to the phase 4 protocol to optimize feasibility.

#### Study Design and Objectives

Phase 3 was a prospective descriptive case series. Participants were recruited and administered the VR-GMI program at the Health Sciences Centre (HSC) in Winnipeg, Canada, a large academic tertiary hospital and level 1 trauma center. The VR-GMI program was administered daily immediately following LLA for a maximum of 10 sessions or until hospital discharge. Quantitative and qualitative self-reported data were gathered pertaining to patient experience, and details about recruitment and retention were documented.

Phase 3 included 2 objectives. The primary objective was to evaluate preliminary recruitment and retention trends as well as identify potential recruitment barriers for phase 4. The secondary objective was to describe participants’ experience with the VR-GMI program in 3 domains, including immersion, tolerability, and overall helpfulness.

#### Recruitment

The research team aimed to recruit 5 to 10 participants by the end of the recruitment period (January 2024 to July 2024), resulting in a sample size of 6 participants. Participants were required to meet the following inclusion criteria: underwent (postoperative) or were about to undergo (preoperative) a unilateral LLA (hip, below knee, above knee, or foot) of either elective or traumatic mechanisms within the last 5 days; currently receiving inpatient treatment at the HSC in Winnipeg, Canada; and fluent in English (speaking and reading). Potential participants were not eligible if they met the following exclusion criteria: any visual, hearing, or motor impairment that would affect engaging with a head-mounted VR headset or hand controls; anyone with or about to undergo a bilateral leg amputation; and anyone aged <18 years. Potential participants were screened from the following avenues: the preanesthetic clinic (which hosts presurgical visits between anesthesiologists and patients before LLA) and elective and emergency operating room slates (which contain the information of every patient about to undergo an LLA). Any LLAs that appeared eligible were recorded. Once potential participants were identified, health care professionals in their circle of care approached them to ascertain their interest in hearing more about the study from research personnel. If interested, research personnel approached the participant at the HSC to ensure they met eligibility criteria and administered the informed consent form. If a patient was ineligible or declined participation, the reason was recorded.

#### VR Intervention Procedure

Participants engaged in 1 VR session per day for approximately 20 minutes from their respective ward (orthopedic, vascular, or rehabilitation). At the beginning of the first VR session, after informed consent was obtained and the honorarium was given to the participant, the research assistant administered the sociodemographic and patient characteristic questionnaire (including age, amputation etiology, place of residence, etc). The research assistant then gave a brief verbal introduction to the VR-GMI system and instructed the participant on how to use the VR headset and handheld controls. Following VR instruction and at the beginning of each subsequent VR session, the research assistant verbally administered the Numerical Rating Scales (NRSs; 11-point Likert scales) for PLP, residual limb pain (RLP; ie, physical pain at the amputation site), and nausea before asking if they wanted to engage with the VR program that day. If they declined participation, their reason was recorded.

Participants were only permitted to engage in 1 stage per day. They could only move onto the next VR stage if their RLP, PLP, or nausea NRS scores did not increase by >2 points during or before their next session (given that treatment may exacerbate pain or nausea, and 3 points is considered a clinically significant increase) [44-47]. The participants were permitted to spend as much time as they preferred in each VR stage. If the participant chose to stop the session before the 20-minute goal (a session length used in previous studies) [23,48], the research assistant asked for and recorded their reason for discontinuation. Afterward, the research assistant verbally readministered the NRSs for PLP, RLP, and nausea. Any impromptu feedback or observations made by research personnel were recorded as field notes.

**Figure 3:**
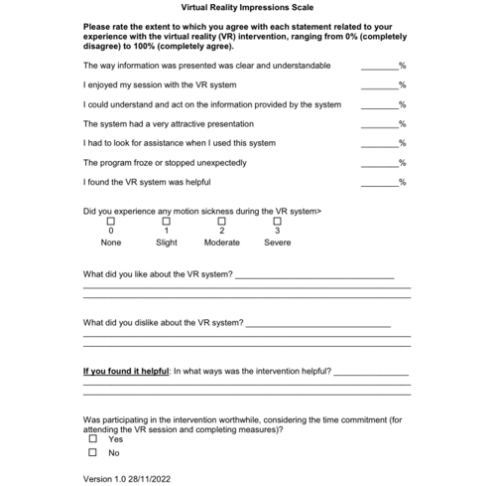
The Virtual Reality Impressions Scale.

Participants were asked to engage in up to 10 VR-GMI sessions or until hospital discharge, whichever came first. After the last VR-GMI session, before discharge, the Igroup Presence Questionnaire (measuring VR immersion) [49] and VR Impressions Scale (developed by the laboratory for the purposes of descriptive evaluation of a VR intervention; Figure 3) [50] were administered.

#### Data Analysis

For the first objective, eligibility and consent rates were calculated according to the number of patients screened. Reasons for refusals or ineligibility were recorded in pie charts. The secondary objective analyzed patient experience with the VR-GMI program by describing the qualitative and quantitative data of each participant individually to capture unique perspectives. Specifically, participant ratings on tolerability (measured by NRS for PLP, RLP, and nausea), immersion (measured by the Igroup Presence Questionnaire), and overall helpfulness (measured by the VR Impressions Scale) were described individually in conjunction with sociodemographic characteristics (such as age, length of hospital stay, etc) to provide context for interpretation. The qualitative data, including participants’ quotes and observations made by research personnel, were integrated to triangulate and increase the strength of the findings.

### Phase 4A: Hospital and Home Feasibility Study (Underway)

#### Overview

In phase 4A, the research team will assess the feasibility and pilot outcomes of the VR-GMI program in the acute postoperative period immediately following LLA (including the hospital and home). The research team aims to recruit 30 participants who recently underwent an LLA through the HSC. A research assistant will visit participants daily to administer the VR-GMI program for approximately 17 days. Participants will then be contacted again 1 month following their final VR session via phone call to assess postoperative PLP. Quantitative data will be collected to assess whether the VR-GMI program and study procedures are sufficient for an RCT.

#### Study Design and Objectives

##### Overview

Phase 4A will consist of a quantitative feasibility study using a 1-group post–test-only design in line with methodological recommendations [33,51]. The feasibility of the program will be evaluated through self-report and administrative quantitative data. The objectives and affiliated hypotheses are mentioned subsequently.

##### Intervention Feasibility

Participants will consider the VR-GMI program to be acceptable, tolerable, and engaging across administration settings (inpatient, outpatient, and home settings) and VR stages.

##### Procedural Feasibility

The used recruitment strategies and study procedures will be appropriate for a RCT, as indicated by comparing sample characteristics to administrative data (reflecting all people undergoing LLAs during the recruitment period) in addition to predetermined recruitment, retention, and attrition rate criteria [52].

##### Pilot Outcomes

Participants who spend an extended amount of time in the VR-GMI program will report lower PLP incidence as well as (for individuals who have already experienced PLP onset) lower intensity and interference. These predictions are in line with previous research investigating prophylactic PLP treatment through mirror therapy in the postoperative period [23].

##### Exploratory Outcomes

The research team will explore reasons for rejecting participation as well as session or study withdrawal. The research team will also investigate whether acceptability, engagement, or PLP incidence and intensity vary according to baseline, sociodemographic, and exploratory variables (such as anxiety, depression, amputation indication, etc).

#### Recruitment

The research team aims to recruit 30 participants, 5 (17%) of whom we expect to drop out and 25 (83%) to complete the program. This sample size is in line with previous pilot studies for PLP treatments that precede RCTs [38] and suits the descriptive and exploratory aims of the study. This sample size also has 80% power to detect the desired recruitment criterion [53].

Potential participants will be approached according to the recruitment strategies and eligibility criteria outlined and recommended in phase 3, with 3 exceptions to help mitigate recruitment barriers faced in the previous phase. First, clinical resource nurses on units where LLAs are treated at the HSC will be called each week to ascertain where eligible patients are to increase the recruitment rate. Second, individuals with bilateral amputations will also be eligible for recruitment, given preliminary data from phase 3 suggesting this may be feasible. Third, patients will be eligible to participate for up to 2 months following their LLA, in line with the literature that suggests such a time frame is suitable for the administration of acute PLP intervention [20].

#### VR Intervention Procedures

**Figure 4:**
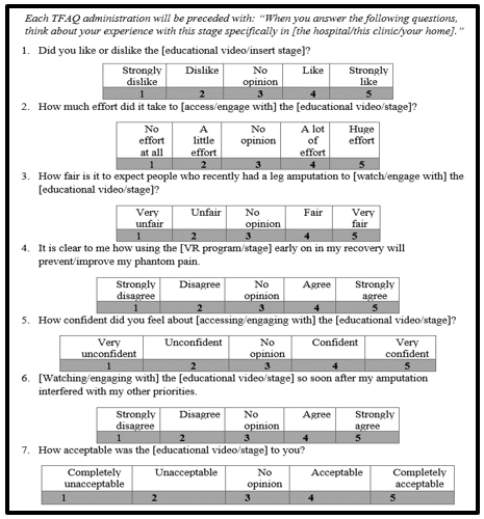
Adapted Theoretical Framework of Acceptability Questionnaire.

Similar procedures to phase 3 will be used in phase 4A, with 3 exceptions made in accordance with preliminary phase 3 data. First, following baseline measure administration, the research assistant will administer a brief video via laptop that introduces the participant to the VR-GMI program, including how to operate the controls and put on the headset. The video (rather than verbal instruction) serves to standardize study expectations and technical knowledge across participants. Second, new measures have been introduced in addition to the measures used in phase 3. The first time a participant completes a stage, they will be administered the Theoretical Framework of Acceptability Questionnaire, which consists of 7 items rated on a 7-point Likert scale inquiring about the acceptability of a behavioral intervention [54] (Figure 4). In addition, following the limb simulation stage (both with and without leg tracking), each participant will be administered the Virtual Embodiment Questionnaire, a validated measure for agency and ownership in a virtual experience involving avatar embodiment [55]. Finally, the Brief Pain Inventory and Patient Reported Outcomes Measurement Information System will be verbally administered over the phone 1 month following each participant’s final VR session to measure PLP intensity and interference, respectively, through validated self-report measures [56-58].

The last and most significant amendment made between phases 3 and 4A is the administration of the VR-GMI program in the home setting following hospital discharge. Data from phase 3 suggested that participants were discharged from the hospital before 2 weeks had passed, which prevented them from receiving the VR dose necessary to see a treatment effect [23]. Therefore, a research assistant will contact participants over the phone following their discharge to inquire about their interest in continuing participation for the remaining sessions in their home. If they wish to continue (and they live in Winnipeg), their participation will proceed with home visits. If they do not reside in Winnipeg, their participation will be discontinued by the researcher; however, their willingness to continue the program will be recorded. Participants will be allowed to continue the VR-GMI program for up to 17 days to analyze long-term acceptability and dosage effects. Immediately following the completion of the feasibility trial, relevant modifications will be made to the prototype based on results.

#### Data Analysis

Objectives 1, 2, and 4 will be evaluated descriptively via bar graphs, means, and SDs to compare each objective across administration settings and VR stage according to preset feasibility criteria. Preset feasibility criteria must be met to determine if the VR program is acceptable (mean Theoretical Framework of Acceptability Questionnaire score >3, which indicates a more favorable acceptability rating than no opinion; Sekhon et al [54]), tolerable (at least 70% of the participants do not report any pre- or post-NRS score increases of ≥3 for PLP, RLP, or nausea), and engaging (mean session duration ≥20 min). Similar progression criteria [52] must be met to determine whether procedural feasibility is appropriate for an RCT, including recruitment (at least 50% of the patients at the HSC who appear to meet the eligibility criteria will be screened through the established pathways), retention (at least 70% of the participants who live in Winnipeg will continue to participate in the study for as long as they are able to), and attrition (at least 80% of the participants will respond at the 1-month follow-up period following their final session) rate criteria. The sample will also be compared to the population of all people who underwent an LLA at the HSC during the recruitment period (from June 2024 to March 2025) via administrative chart review to evaluate whether the characteristics are relatively proportional to one another.

For objective 3, scatterplots will be generated to explore the association between the length of program engagement and the Brief Pain Inventory or Patient Reported Outcomes Measurement Information System pain interference. Mean or median difference in length of program engagement between participants who have and have not yet experienced PLP onset will also be calculated. If appropriate, exploratory regression models will be used to examine the effect of VR dosage on PLP outcomes.

### Phase 4B: Focus Groups

Phase 4B will conclude with a series of focus groups (n=2) consisting of a subset of 5-10 interested participants from phase 4A following completion. Participants will meet in person to discuss their experiences with the VR GMI program in the hospital and home (if applicable), facilitated by members of the research team using a semistructured interview. Immediately following the initial discussion, participants will be invited to trial the updated prototype (headsets will be circulated, and snacks will be provided to those waiting for their turns). Following the trial, another group discussion will occur to provide feedback on the new developments, also using a semistructured interview format. The focus group transcripts will then be analyzed through reflexive thematic analysis [42,43] and interpreted to inform the final development of the VR GMI program.

## Results

Initial funding for prototype development and phase 1 was obtained through the NRC New Beginnings Fund, awarded to REG and MSDS as co–principal investigators in December 2020 (US $25,000 + 20% of full-time employment [or 390 h] of engineering time for development). Delays occurred in the initial development of the program due to the COVID-19 pandemic and parental leave (REG). Phase 1 of this study was completed in January 2023, at which point recruitment for phase 2 was conducted until September 2023 and is now published [59]. Additional funding was obtained through the Winnipeg Foundation Innovation Fund (CAD $100,000 [US $74,088] to principal investigator REG) in December 2023. Recruitment for phase 3 began in January 2024 and was completed in July 2024. As indicated earlier, several methodological modifications were conducted to the study protocol before initiation of the final phase. Phase 4A commenced in August 2024 and is expected to be completed in June 2025. As of March 2025, we have screened 101 patients for eligibility and successfully recruited 15 participants. Regardless of ethics approval to initiate intervention before surgery, hospital logistics and communications have created challenges around recruiting participants preoperatively. Phase 4B will be conducted in June 2025, followed by the final refinements of the VR-GMI program. Should significant adjustments need to be made to the program, an additional feasibility trial will be conducted before the larger clinical trial. It is anticipated that results from phase 4 will be published by January 2026.

**Figure 5:**
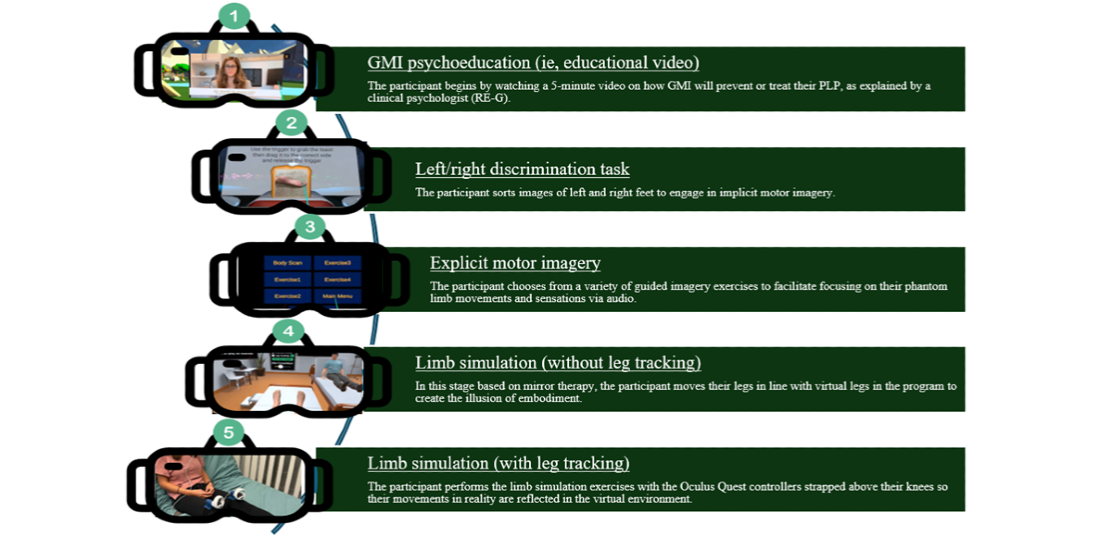
The 5 stages of the virtual reality–graded motor imagery program (before entering phase 4A). GMI: graded motor imagery; PLP: phantom limb pain.

Meanwhile, the research team will use preliminary data to inform iterative development of the VR-GMI program; between studies, these developments are deployed so that each phase evaluates a higher fidelity prototype than the last. For example, information from phase 2 suggested that a video was embedded into the VR-GMI program to educate patients on the potential benefits of using this treatment. Moreover, VR controls have continuously been simplified to promote treatment use by individuals with lower technological self-efficacy (eg, allowing them to use only 1 control to operate the VR GMI program). Most notably, results from phase 2 indicated that better immersion may be experienced in the VR-GMI program via the use of hand controls as leg trackers. Leg trackers were then 3D printed to allow trial in phase 3. Detailed results from all phases will be available in a future paper to be published. Figure 5 gives an overview of the VR-GMI prototype in its current state, given preliminary data collection and analysis.

## Discussion

### Comparison With Prior Work

This protocol represents both a novel methodological approach and an intervention through which PLP in people with LLAs can be treated. Despite the success VR and GMI have each demonstrated as independent PLP treatments [17,24], they have never been combined. Beyond its potential to increase GMI efficacy, VR can be used to streamline all the GMI components into a convenient, commercially available VR program, improving patient access both economically and logistically. Previous interventions have also leveraged this unique advantage of VR to administer PLP treatment in the home, often using gamification [28,29,60]. However, this is the first protocol to deliberately tailor a VR PLP program to the postoperative acute care setting. Moreover, while previous studies have demonstrated the prophylactic capacity of mirror therapy [20,23], no research has focused on how VR might enhance patient engagement and improve administration efficiency to reduce or prevent PLP onset. These novel contributions are noteworthy strengths that could create a conceptual foundation for further inquiry.

### Strengths and Limitations

The most significant strength of this protocol is its prioritization of feasibility through a user-centered approach before empirically assessing the efficacy of the VR-GMI intervention in a clinical trial design. According to the VR-CORE framework [33], using patient-centered design is essential to the iterative development of an effective VR health care intervention. Recently, there has been a movement to design VR interventions with explicit consideration of implementation outcomes (such as acceptability) from the beginning of development [61]. Despite this recommendation, very few studies have examined the feasibility of VR PLP interventions in ecologically valid settings. Many researchers instead develop VR PLP interventions solely to assess their capacity for short-term PLP reduction [62,63]. Such inadequate focus on the feasibility of VR PLP interventions in target settings may contribute to low treatment adherence, limiting and confounding their effectiveness [64]. This 4-phase protocol attempts to address this common issue in VR literature with a thorough consideration of feasibility before conducting an RCT, serving as an example for the application of the VR-CORE framework.

However, each phase of the protocol also presents limitations. The feedback collected in phase 1 was collected informally through open-ended questions; therefore, the resulting recommendations were the result of simple compilation rather than systematic analysis. Furthermore, due to the COVID-19 pandemic, only 2 study partners with lived PLP experience were available to trial the VR prototype in person (all were able to observe video developments). Furthermore, although the initial goal was to recruit 5 diverse partners with lived PLP experience, only 3 were consulted. Meanwhile, phase 2 recruitment was completed via convenience sampling and was limited to interested individuals able to attend at the NRC building. Thus, the results of phase 2 likely do not capture the barriers encountered by the diverse population undergoing LLAs in Canada. Phases 3 and 4 have and will encounter similar bias. While a more representative sample may be recruited at the outset, data from phase 3 suggest participants are discharged from the hospital before completing the VR program for the recommended treatment period. Because only individuals who live in Winnipeg may logistically continue the program after discharge, people who live in rural or Northern communities are unlikely to receive the VR dosage necessary to demonstrate a treatment effect. This exclusion limits generalizability to Indigenous peoples living in Northern communities, who are disproportionately affected by diabetic LLA [65]. These limitations highlight that, while we can use the data of this protocol to inform VR development, more representative recruitment will be necessary before the clinical feasibility of the program for the general population can be ascertained.

### Future Directions

Beyond this protocol, further development of the VR-GMI program will prioritize clarifying its feasibility and evaluating its efficacy in the postoperative acute period. Depending on the results of phase 4A, additional feasibility studies on the VR-GMI program may be necessary. This may include a longitudinal study conducted in the homes of participants beyond the city limits of Winnipeg to capture a more representative sample. Furthermore, an RCT will be conducted to assess the efficacy of the program. The first RCT will compare virtual GMI to a distractor control (ie, a natural VR environment), while another RCT may compare the program to traditional GMI. The studies enhance the development of an effective VR program to prepare for clinical implementation. Dates for these studies have not yet been determined as initiation depends upon the results of phases 4A and 4B.

The research team also aims to promote visibility of the proposed intervention through brand awareness to spark interest in its use by health care professionals. In line with this aim, the VR-GMI program will henceforth be coined PIVOT (Phantom Pain Graded Motor Imagery Through Virtual Reality Orthopedic Training). PIVOT will be referenced in upcoming publications, which will detail the results of phases 2, 3, and 4. While a name is ultimately a very small change, it is a step toward connecting with health care professionals that may assist with the clinical implementation of PIVOT following prototype development.

### Conclusions

This protocol represents a novel methodological approach to iteratively develop a patient-centered VR intervention. Moreover, it outlines the development of the PIVOT program, which could increase the effectiveness and accessibility of PLP treatment through engaging and prompt self-administration of GMI. Following rigorous feasibility and efficacy evaluation, the clinical implementation of the PIVOT program could improve the quality of life of millions of people with LLAs by reducing their PLP and its downstream negative sequelae.

## Appendix

Multimedia Appendix 1 Development recommendations from study partners with lived experience with amputations.

## Abbreviations

GMI: graded motor imagery
HSC: Health Sciences Centre
LLA: lower limb amputation
NRC: National Research Council
NRS: Numerical Rating Scale
PIVOT: Phantom Pain Graded Motor Imagery Through Virtual Reality Orthopedic Training
PLP: phantom limb pain
RCT: randomized controlled trial
RLP: residual limb pain
VR: virtual reality
VR-CORE: Virtual Reality Clinical Outcomes Research Experts

## Data Availability

The datasets generated or analyzed during this study are not publicly available due to small sample sizes and the collection of qualitative data but may be available from the corresponding author on reasonable request.
