# Supplementary material for: Treating Lower Phantom Limb Pain in the Postoperative Acute Care Setting Using Virtual Reality: Protocol for a 4-Phase Development and Feasibility Trial
Source: JMIR Res Protoc. 2025 May 23;14:e68008. doi: 10.2196/68008 (PMC12144477; doi:10.2196/68008)
Supplement: Multimedia Appendix 1 [file resprot_v14i1e68008_app1.docx]

## Multimedia Appendix

Development recommendations from study partners with lived experience.

1. **Address Technological Barriers.** Both patient partners and physiotherapists remarked that the VR program would likely garner more interest from younger individuals, given the age gap in technological familiarity. Therefore, technical instructions were added to the study protocol and limb simulation was simplified for ease of use.
2. **Optimize Grading.** Physiotherapists emphasized the importance of standardizing when and how patients should progress onto a new stage of the VR program during long-term use. Progressing through the GMI too quickly could result in temporarily worsened PLP, also known as a flare-up. To mitigate this risk, partnered physiotherapists recommended continuously monitoring the pain intensity of patients via NRS to ensure a participant does not experience a clinically significant NRS increase in any given session (i.e., an increase of 3 or more).
3. **Optimize Leg Tracking.** Leg tracking in the limb simulation stage garnered mixed reactions from the patient partners and physiotherapists. One patient partner strongly advocated for the leg trackers, stating it significantly improved their sense of agency in the virtual legs. However, physiotherapists thought the leg tracking should only be applied to some of the exercises, given they were only of immediate benefit to exercises that involved large movements of the leg with straight knees (i.e., leg lift and hip adduction exercises). Eventually, leg trackers may be placed both above and below the knee to allow for knee bending in the VR program. Feedback also indicated that the leg trackers were not working properly, with the virtual legs often interacting with the environment in unrealistic ways. This resulted in developing new leg trackers that involved attaching the hand controllers to the legs to facilitate more accurate visual feedback in Phase 2.
4. **Improve Hospital Feasibility.** Our study partners suggested a variety of factors that could affect one’s ability to engage in the VR program in hospital immediately following their amputation surgery. For example, sensitivity of the residual limb could make it difficult to engage in the mirror therapy stage mentally or physically, such as putting on the leg trackers or differentiating between phantom and physical phantom limb pain. Accordingly, they suggest the VR program should not be administered immediately following amputation surgery—at least two days should be given so the patient can acclimate to limb loss.
5. **Peer Support.** Many study partners also noted that it would have been useful to have more knowledge about the rehabilitation process prior to their amputation surgeries. For example, being introduced to someone who had successfully adapted to life with their amputation would have lowered their anxiety and skepticism about surgery and subsequent treatment, respectively. This feature could potentially be implemented in future stages of development.
